# TCP Transcription Factors in Moso Bamboo (*Phyllostachys edulis*): Genome-Wide Identification and Expression Analysis

**DOI:** 10.3389/fpls.2018.01263

**Published:** 2018-10-05

**Authors:** Huan-Long Liu, Min Wu, Fei Li, Ya-Meng Gao, Feng Chen, Yan Xiang

**Affiliations:** ^1^National Engineering Laboratory of Crop Stress Resistance Breeding, School of Life Sciences, Anhui Agricultural University, Hefei, China; ^2^Laboratory of Modern Biotechnology, School of Forestry and Landscape Architecture, Anhui Agricultural University, Hefei, China

**Keywords:** moso bamboo (*Phyllostachys edulis*), TCP transcription factors, expression patterns, subcellular localization, transcription activity

## Abstract

TEOSINTE BRANCHED 1, CYCLOIDEA, and PROLIFERATING CELL FACTORS (T), members of a plant-specific gene family, play significant roles during plant growth and development, as well as in response to environmental stress. However, knowledge about this family in moso bamboo (*Phyllostachys edulis*) is limited. Therefore, in this study, the first genome-wide identification, classification, characterization, and expression pattern analysis of the TCP transcription factor family in moso bamboo was performed. Sixteen TCP members were identified from the moso bamboo genome using a BLASTP algorithm-based method and verified using the Pfam database. Based on a multiple-sequence alignment, the members were divided into two subfamilies, and members of the same family shared highly conserved motif structures. Subcellular localization and transactivation activity analyses of four selected genes revealed that they were nuclear localized and had self-activation activities. Additionally, the expression levels of several PeTCP members were significantly upregulated under abscisic acid, methyl jasmonate, and salicylic acid treatments, indicating that they play crucial plant hormone transduction roles in the processes of plant growth and development, as well as in responses to environmental stresses. Thus, the current study provides previously lacking information on the TCP family in moso bamboo and reveals the potential functions of this gene family in growth and development.

## Introduction

Plants encounter a variety of environmental stresses during their growth and development; therefore, they possess various protective systems at the whole-plant, tissue, cellular, subcellular, genetic, and molecular levels (Sheshadri et al., 2016). Stress-inducible genes are the major molecular factors involved in environmental stress responses and increased tolerance (Yamaguchishinozaki and Shinozaki, 2005). These genes have generally been divided into two categories, one involved directly in stress tolerance and the other in signal transduction and transcriptional regulation. Transcription factors (TFs) are important groups of regulatory genes (Wu et al., 2015). A series of TFs that are involved in governing developmental responses to the environment have been identified and reported in plants, such as MYB (salt and drought tolerance) (Zhang et al., 2012; Cui et al., 2013), ERF (ethylene responsive factors) (Lata et al., 2014), WRKY (regulation of stress response) (He et al., 2012; Yan et al., 2014; Cai et al., 2017), HD-Zip (drought and salt tolerance) (Zhao et al., 2011, 2014), and bZIP (pathogen defense regulation) (Sheshadri et al., 2016). In addition, a previous study reported that TCP TFs directly translate environmental signals to cope with environmental stresses during plant growth (Danisman, 2016).

TCPs, a family of plant-specific TFs (Martíntrillo and Cubas, 2010), were named based on the first three identified members, maize teosinte branched1 (*TB1*), *Antirrhinum* cycloidea (*CYC*), and rice PCF proteins (*PCF*s) (Doebley et al., 1997; Kosugi and Ohashi, 1997; Cubas et al., 1999; Luo et al., 1999). The TCPs contain a distinguished domain with a non-canonical basic helix–loop–helix structure based on a sequence alignment analysis of TCP proteins, namely TCP domain (Doebley et al., 1997; Kosugi and Ohashi, 1997; Cubas et al., 1999; Luo et al., 1999). Interestingly, most of the members of the ECE clade contain a conserved R domain that might be involved in protein–protein interactions (Cubas et al., 1999), while some members of the CIN clade independently acquired an R domain (Cubas, 2002).

At present, many functional analyses of TCPs have been reported in various plant developmental processes. For example, TCPs play essential roles during the morphogenetic period in plants. TB1 and TB1-like proteins from maize, rice, sorghum, and Arabidopsis repress the development of axillary branches (Hubbard et al., 2002; Takeda et al., 2003; Kebrom et al., 2006; Aguilar-Martínez et al., 2007; Finlayson, 2007). In Arabidopsis, some members are involved in leaf development. For example, *TCP14* and *TCP15* interact with the ubiquitin receptors DA1, DAR1, and DAR2, which regulate internode length and leaf shape by repressing endoreduplication (Peng et al., 2015). Five members (*TCP2, TCP3, TCP4, TCP10*, and *TCP24*) have been implicated in the regulation of leaf morphogenesis (Schommer et al., 2008). Additionally, *AtTCP20*, acting upstream of *AtTCP9*, controls leaf development through the jasmonate signaling pathway. *GhCYC2*, a TCP member in chrysanthemum, regulates the development of petals (Broholm et al., 2008). In *Pisum sativum*, two CYC-like TCP proteins control floral zygomorphy (Wang et al., 2008). In cotton, TCPs play positive roles in fiber elongation (Hao et al., 2012). In addition, TCP proteins might play important roles during the generative growth phase, such as in the development and maturation of fruits (Parapunova et al., 2014). *TCPs* not only contribute to plant morphogenetics, they also mediate the regulation of rhythms. For instance, TCP proteins can regulate the circadian clock in *Arabidopsis thaliana* (Giraud et al., 2010) through binding to the TGGGC(C/T) elements. Furthermore, TCP TFs contribute to plant resistance to diverse abiotic stresses. For example, *OsPCF5* is involved in drought- and salinity-stress tolerance, and *OsPCF6* takes part in cold-stress tolerance in rice (Wang et al., 2014). *OsTCP15* plays a part in the mesocotyl elongation response to darkness in rice (Hu et al., 2014). *TCP20* can interact with *NLP6&7* and support root meristem growth under N starvation conditions in *Arabidopsis thaliana* (Guan et al., 2017). *OsPCF2* activates *OsNHX1* expression and enhances its salt tolerance (Almeida et al., 2016). Furthermore, *OsPCF6* and *OsTCP21*, identified as target genes of Osa-miR319b, are involved in cold-stress tolerance (Wang et al., 2014).

Moso bamboo with high ecological, cultural, and economic values is faced with various environmental stresses in the course of its growth and development. This requires moso bamboo to respond and resist adverse conditions in a timely manner. Genomic studies in bamboo, including genome-wide full-length cDNA sequencing (Peng et al., 2010), chloroplast genome sequencing (Zhang et al., 2011), identification of syntenic genes between bamboo and other grasses (Gui et al., 2010), phylogenetic analysis of Bambusoideae subspecies (Sungkaew et al., 2009), and the construction of the draft genome sequence of moso bamboo, laid the foundation for researching and improving stress resistance at the genetic level. So far, various gene families having potential resistance functions in moso bamboo have been analyzed, such as the WRKY TF family (Li et al., 2017; Wu M. et al., 2017), homeodomain leucine zipper subfamily (Chen et al., 2017), and AP2/ERF TF family (Wu et al., 2015). The study of TCP TFs remains enigmatic, even though they play significant roles in plant development and growth as well as in stress resistance. In the present study, we searched the moso bamboo genome to identify the genes encoding TCP TFs (PeTCPs). In total, 16 *PeTCP*s were identified from the moso bamboo genome. The characteristics of these genes, including structure, phylogenetic relationship, promoter elements, evolution divergence, subcellular localization, transactivation activity, and expression patterns, were subsequently identified.

## Materials and Methods

### Identification of TCP Genes

To obtain comprehensive and non-redundant moso bamboo proteins containing the TCP domain, a BLASTP algorithm-based search provided by the BambooGDB database^1^ was first performed using the seed sequences of reported rice TCP proteins (Yao et al., 2007; Mondragónpalomino and Trontin, 2011; Dhaka et al., 2017). The default parameters were adopted, and the cutoff value was set to 0.01. The obtained putative TCP proteins were subsequently removed manually and further verified using the Pfam^2^ database (Finn et al., 2006, 2010). Detailed information on each putative TCP protein, coding sequences (CDSs), and amino acid lengths, as well as physicochemical parameters, were obtained from the BambooGDB database. Subcellular localizations of PeTCPs were predicted using ProtComp 9.0. Gene IDs of rice, *Brachypodium distachyon, Arabidopsis thaliana*, poplar, and *Sorghum* were obtained from earlier reports (Yao et al., 2007; Francis et al., 2016; Ma et al., 2016) and their sequences were downloaded from TIGR^3^, BioMart^4^, TAIR^5^, and Phytozome^6^ databases, respectively.

### Phylogenetic and Multiple Sequence Alignment Analysis

To study the phylogenetic relationships of *TCP* genes among different species, Clustal X 2.0 (Larkin et al., 2007) was used to perform multiple sequence alignments, and MEGA 6.0 (Tamura et al., 2013) was subsequently used to construct a phylogenetic tree based on the multiple alignment results by using the neighbor-joining method (parameters: 1,000 bootstraps).

### Gene Structure and Conserved Motif Analyses

To investigate the exon/intron structures of the *TCP* genes, the Gene Structure Display Server was used with the corresponding CDSs and genomic DNA sequences on default parameter settings. The online tool MEME^7^ was used to identify and analyze the conserved motifs of TCP proteins (parameter setting: maximum number of motifs, 10; maximum width, 100).

#### Synonymous (*K*a) to Non-synonymous (*K*s) Mutation Ratio Analyses Among Moso Bamboo, Rice, Maize, and *Brachypodium distachyon*

*K*a and *K*s were computed using DnaSP 5 software (Librado and Rozas, 2009) based on the pairwise alignment of homologous pairs between moso bamboo and other grass species using MEGA 6.0. The divergence time (T) was calculated using the formula *T* = *K*s/2λ (λ = 6.5 × 10^-9^) (Peng et al., 2013).

#### Putative Promoter *cis*-Acting Element Analysis

The 2,000-bp upstream sequences of the *PeTCP* gene sequences were submitted to PlantCARE (Lescot et al., 2002)^8^ to predict putative promoter *cis*-acting elements.

#### Plant Material Growth Conditions and Stress Treatments

Three-month-old seedlings were germinated and grown in an artificial growth chamber (planted in a flowerpot with a diameter of approximately 20 cm) with a 16-h light/8-h dark cycle at 22°C. These seeds had been collected in the Tianmu Mountain National Nature Reserve in Zhejiang Province, China. To analyze the tissue expression patterns of *TCP* genes, samples of six tissues, including mature leaves, young leaves, stems, shoots, rhizomes, and roots, were collected. To investigate the expression patterns of *TCP* genes under stress-related plant hormone treatments, seedlings were sprayed with 0.1 mM abscisic acid (ABA), 0.1 mM methyl jasmonate (Me-JA), or 1 mM salicylic acid (SA) treatments. Additionally, control samples were similarly sprayed with distilled water. For each stress treatment, all of the samples were collected at six time points (0, 1, 3, 6, 12, and 24 h). All plant samples were immediately stored in a -80°C freezer after collection for RNA extraction. Three repeated trials and three biological replicates were performed for each sample.

#### Transactivation Activity Analysis in Yeast

The pGBKT7 vector (Clontech, Palo Alto, CA, United States) was used to study the transcriptional activity levels of four PeTCP proteins in yeast. The full-length PeTCPs open reading frames were PCR amplified and independently cloned into the pGBKT7 vector (Clontech; containing the GAL4 DNA-binding domain) using the gene-specific primers listed in **Supplementary Table S5**. Subsequently, pGBKT7-PeTCP recombinant vectors, the positive control pGBKT7-53+pGADT7-T, and the negative control pGBKT7 empty plasmids were used to transform the yeast strain using the lithium acetate method. The transformed strains were further serially cultured on various SD selective media, including SD/-Trp and SD/-Trp/-His/-Ade/X-α-Gal, and incubated at 30°C for 3–5 days.

#### qRT-PCR Analysis

The qRT-PCR experiment was used to explore the expression levels of TCP members in different tissues or developmental stages, and under abiotic stress and plant hormone treatments. Total RNA was extracted from the plant samples using TRIzol and cetrimonium bromide methods and was then reverse transcribed into cDNA using a PrimeScript^TM^ RT Reagent Kit (TaKaRa, Dalian, China). Gene-specific primers were designed using Primer Express 3.0, and tonoplast intrinsic protein 41 (TIP41) was used as an internal control (Fan et al., 2013). TransStart^®^ Tip Green qPCR Super Mix (TransGen Biotech, Beijing, China) was used in the qRT-PCR master mix and the program was run as specified in the instructions.

### Statistical Analysis

Statistical significance was determined using a paired Student’s *t*-test^9^. The mean ± standard deviations from the mean (SD) of at least three replicates are presented, and significant differences relative to controls are indicated at ^∗^*p* < 0.05 and ^∗∗^*p* < 0.01.

## Results

### Identification and Characterization of TCP TFs

Prospective TCP members from the moso bamboo genome were obtained from BambooGDB using a BLASTP algorithm-based search with seed sequences from reported rice TCP proteins. A total of 16 TCPs, named *PeTCP1*–*16* based on their physical locations on the scaffold (**Table 1**), were subsequently identified after confirming the presence of the conserved TCP DNA-binding domain (PF03634) using the Pfam database and deleting redundant sequences manually based on the results of a sequence alignment performed using Clustal X. The detailed characteristics of the PeTCPs, consisting of gene name, accession number, locational information, and physicochemical parameters, are provided in **Table 1**. The CDS lengths ranged from 303 to 1,221 bp, resulting in amino acid sequences ranging from 100 to 406 aa. The molecular weight ranged from 10.40 to 43.37 kDa, and the theoretical isoelectric point varied from 4.78 to 10.76. In addition, the locational information of *PeTCP* genes revealed that the 16 *TCP* genes were distributed in different scaffolds, with none of the genes being located on the same scaffold (**Table 1**).

**Table 1:** Detailed information about *TCP* genes in moso bamboo genome.

				Protein			
					
Name	Gene ID	Location	CDS (bp)	Size (aa)	MW(kDa)	pI	Subcellular localization
*PeTCP1*	PH01000001G3420	2225231–2227702(-)	597	198	21.02	10.76	Nuclear
*PeTCP2*	PH01000018G1460	906436–909312(+)	1221	406	43.37	7.02	Nuclear
*PeTCP3*	PH01000028G2500	1606492–1608084(-)	588	195	20.56	10.11	Nuclear
*PeTCP4*	PH01000034G2110	1532558–1534930(+)	1128	375	38.02	9.42	Nuclear
*PeTCP5*	PH01000065G1920	1192667–1194687(-)	1014	337	34.68	8.95	–
*PeTCP6*	PH01000099G0140	103728–105193(-)	867	288	31.11	6.53	–
*PeTCP7*	PH01000131G1060	662980–665991(-)	636	211	21.79	4.85	Nuclear
*PeTCP8*	PH01000135G0620	453977–455750(-)	732	243	24.02	7.87	Nuclear
*PeTCP9*	PH01000155G0570	362966–364770(-)	714	237	26.21	7.14	–
*PeTCP10*	PH01000256G0700	548272–549952(-)	462	153	16.06	10.70	Nuclear
*PeTCP11*	PH01000423G0070	41444–42633(-)	828	275	29.60	7.24	–
*PeTCP12*	PH01000519G0700	449323-453277(+)	972	323	33.70	9.76	–
*PeTCP13*	PH01000602G0590	389150–391333(-)	873	290	32.23	9.20	Nuclear
*PeTCP14*	PH01000767G0040	36399–38799(+)	1110	369	38.50	9.23	Nuclear
*PeTCP15*	PH01001418G0330	226485–230958(-)	456	151	16.58	9.51	–
*PeTCP16*	PH01001480G0290	228472–230034(-)	735	244	25.11	5.55	Nuclear


**FIGURE 1:**
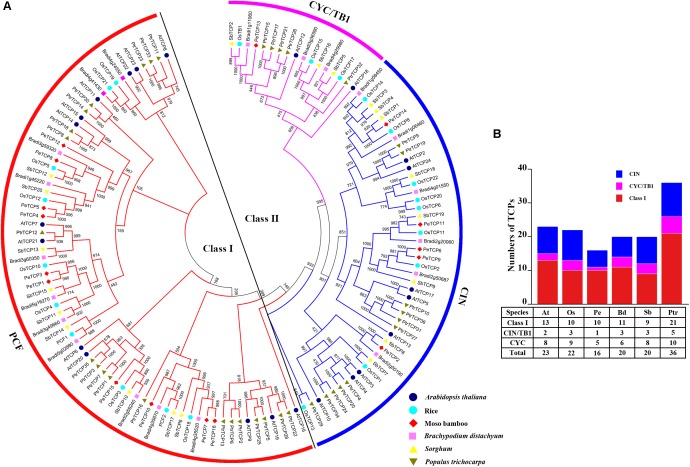
Phylogenetic analysis and statistical analysis of TCP members in different species. **(A)** Phylogenetic analysis of TCP members from moso bamboo, rice, *Brachypodium distachyon, Sorghum, Arabidopsis thaliana*, and poplar. Geometric figures of different colors and shapes are used to mark the TCP members from different species. **(B)** Statistical analysis of TCP members from moso bamboo, rice, *Brachypodium distachyon, Sorghum, Arabidopsis thaliana*, and poplar.

To explore the phylogenetic relationship of TCP TFs among different species and investigate the potential function of PeTCP TFs compared with other well-studied TCP members, a phylogenetic tree was constructed using the neighbor-joining method. The tree was based on the sequence alignment and analysis of 137 full-length amino acid sequences from moso bamboo, rice (Yao et al., 2007), *Brachypodium distachyon*, sorghum (Francis et al., 2016), *Arabidopsis thaliana* (Yao et al., 2007), and poplar (Ma et al., 2016; **Figure 1A**). The information on *TCPs* was obtained from previous studies (**Supplementary Table S1**). TCPs can be divided into two distinct classes: Class I and Class II. Class I was named the PCF group (Cubas, 2002). Class II was further divided into two groups: CIN and CYC/TB1. In moso bamboo, the number of TCPs identified as PCF, CIN, and CYC/TB1 protein members were 10, 5, and 1, respectively. The counting of TCP TFs among the six species showed that the maximum and minimum numbers of TCPs were found in poplar and moso bamboo, respectively (**Figure 1B**). The CYC/TB1 group is the smallest.

### Multiple Sequences Alignment

**FIGURE 2:**
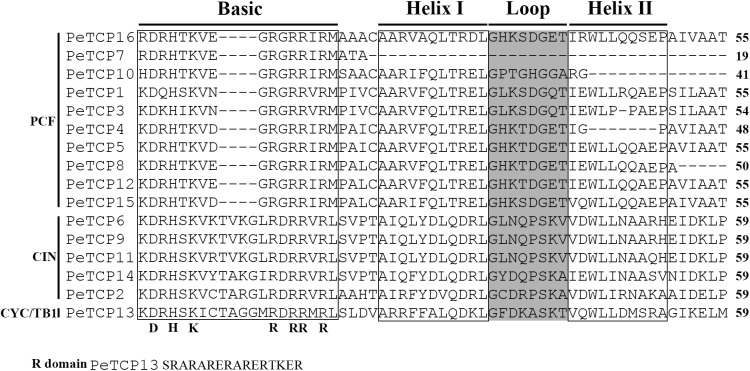
Multiple sequence analysis of TCP domain. Multiple sequence alignment was carried out with Clustal X 2.0.

To investigate the conservation and diversity of the TCP domain regions in TCP proteins, a multiple sequences alignment was performed using Clustal X software based on the amino acid sequences of each TCP domain (**Figure 2**). Alignment results showed that differences and similarities coexisted among these members. For example, complete basic helix I–loop–helix II regions could be found in all TCP members of Class II, resulting in the conservation of this group. In contrast, TCP members of Class I displayed a higher diversity level. For example, four genes, *PeTCP4, PeTCP7, PeTCP8*, and *PeTCP10*, contained only partial TCP domains. In addition, TCP members of Class II had 4-amino acid insertions compared with Class I in the basic regions. In addition, the arginine-rich R domain was found in the C-terminus of *PeTCP13*.

### Gene Structures and Conserved Motifs Analysis

**FIGURE 3:**
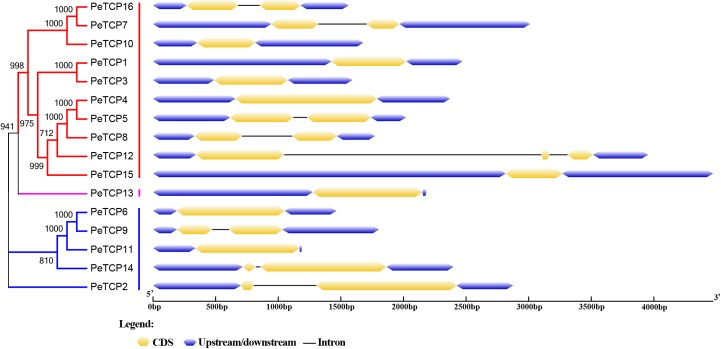
Gene structures of TCPs in moso bamboo. Gene structures were performed using the GSDS online tool. Exons, introns, and untranslated regions (UTRs) are indicated by yellow rectangles, gray lines, and blue rectangles, respectively.

**FIGURE 4:**
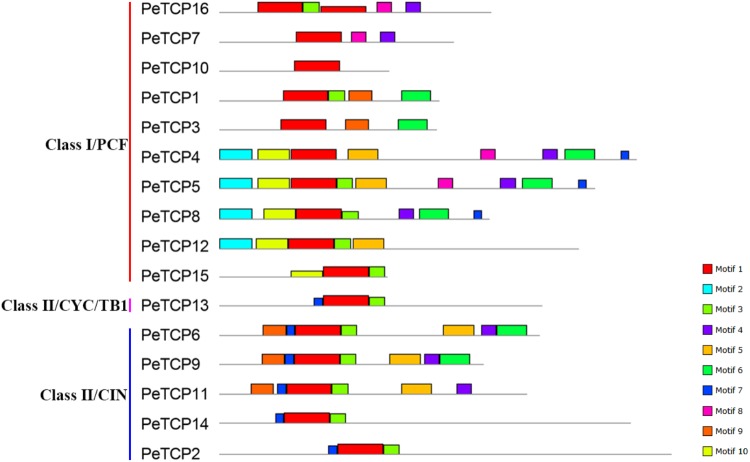
Schematic representation of the 10 conserved motifs in PeTCPs. Conserved motifs of the PeTCPs were identified using the online MEME program based on 16 full-length amino acid sequences with the following parameters: maximum number of motifs, 10; maximum width, 100. The lengths and positions of different motifs in the protein sequences are identified by the lengths and positions of the different color blocks.

The Gene Structures Display Server online tool was used to explore the exon/intron structures of *PeTCP* genes (**Figure 3**) based on the CDSs and the corresponding genomic DNA sequences of the *PeTCP* genes. Of these, 10 *PeTCP* genes lacked an intron, and the remaining members of the *PeTCP* genes only had one or two introns. This result was consistent with previous reports. As shown in **Figure 2**, the online MEME tool (parameter setting: maximum number of motifs, 10; maximum width, 100) was used to predict the conserved PeTCP protein motifs by submitting full-length amino acid sequences and identifying 10 specific motifs (**Figure 4** and **Supplementary Table S2**). The sequence of each motif was verified using the Pfam database and only one motif (motif 1) encoded the TCP domains. The remaining motifs did not encode any domains. Motif 1 was present in every moso bamboo TCP protein we verified, providing further support for the reliability of our identification. Among the PeTCPs, the number of motifs varied from one (*PeTCP10*) to nine (*PeTCP5*). Some motifs were specific to particular members. For example, motif 2 existed only in *PeTCP4, 5, 8*, and *12*; motif 10 was present only in *PeTCP4, 5, 8, 12*, and *15*; and motif 8 only appeared in *PeTCP4, 5, 7*, and *16*. These three motifs existed in Class I with a high specificity. Furthermore, all the members of the Class II *PeTCP*s were characterized by motif 7 in the N-terminal TCP domain. By comparison, the C-terminal TCP domain of motif 3 was detected in various PeTCP members that were widely distributed in the TCP family, except four *PeTCP* genes (*PeTCP3, 4, 7*, and *10*).

### Putative Promoter *cis*-Acting Element Analysis of Stress-Related Plant Hormone

*cis*-Elements are linear nucleotide fragments of non-coding DNA (Biłas et al., 2016). They are present in promoter regions and other transcribed DNA strands (Vaughn et al., 2012). The promoter regions of stress-inducible genes contain *cis*-elements that directly influence the gene regulation involved in stress-responsive gene expression (Yamaguchishinozaki and Shinozaki, 2005). Many stress-responsive genes that encode TFs, with functions of signal transduction and transcriptional regulation, play significant roles in environmental stress tolerance (Yamaguchishinozaki and Shinozaki, 2005; Sheshadri et al., 2016). Various TFs interact with *cis*-acting elements in promoter regions and form transcriptional initiation complexes on the TATA box (core promoter) upstream of the transcriptional initiation site. The transcriptional initiation complex then activates RNA polymerase to start the transcription of stress-responsive genes.

**FIGURE 5:**
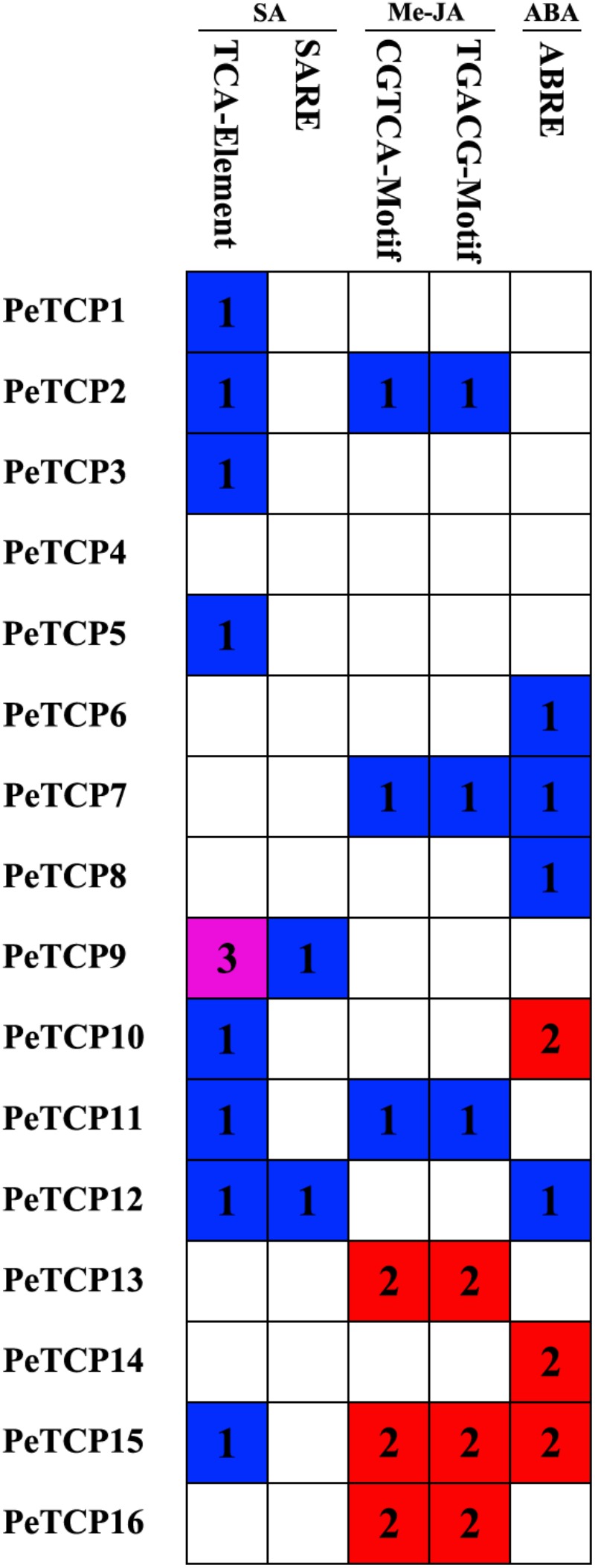
*cis*-Acting elements related to ABA, Me-JA, and SA in the promoter regions of PeTCPs. A *colored block* with a number represents the *cis*-element number of PeTCPs.

When plants are under stressed conditions, TF binding to *cis-*elements is the major genetic level change occurring in the plants. Hence, identifying the *cis*-acting elements in the stress-responsive promoters is important to understand the molecular switches affected by stress-inducible genes. In this study, to explore the *cis-*element patterns and types of *PeTCP* genes, the *cis-*elements in the 2,000 bp upstream of the promoter regions of 16 *PeTCP* genes were predicted using the PlantCARE database. Many types of *cis*-elements involved in environmental stress responses were identified, including ABA-, Me-JA-, and SA-responsive elements (**Figure 5**). The TCA-element (Goldsbrough et al., 1993) and SARE motif associated with SA responsiveness were present in nine *PeTCP* genes (*PeTCP1–3, 5, 9–12*, and *15*). Interestingly, the Me-JA-responsive elements, CGTCA and TGACG, existed in pairs in six *PeTCP*s (*PeTCP2, 7, 11, 13, 15*, and *16*). The ABA-responsive elements (ABRE *cis*-acting element) (Shen and Ho, 1995) were found in the promoter regions of *PeTCP6–8, 10, 12, 14*, and *15*. Among these *TCP* genes and environmental stress-responsive elements, we found that *PeTCP9* and *PeTCP14* had single types of responsive elements, SA-responsive elements and ABAREs, respectively. The other members presented more than one type of stress-responsive elements, such as four types in *PeTCP12* and three types in *PeTCP15.*

### Selection Pressure and Divergence of *TCPs* Between Moso Bamboo and Three Other Grass Species

**FIGURE 6:**
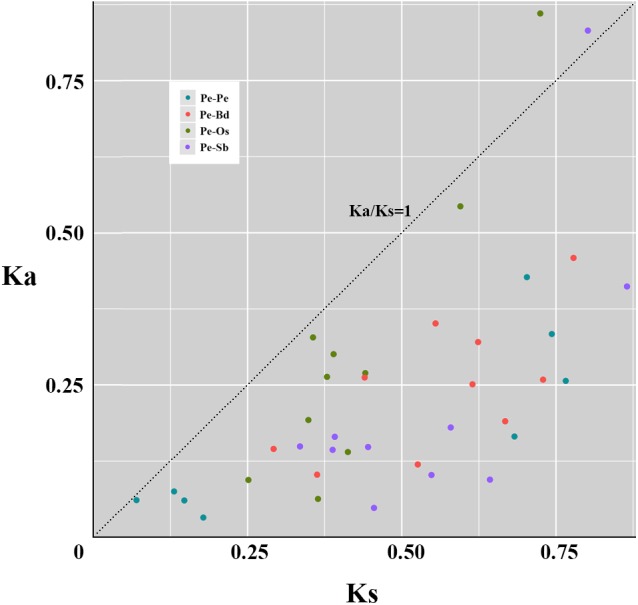
Scatter plot statistics of *K*a and *K*s values among grass species. The black dotted line with slope one is used to show *K*a/*K*s = 1. A circle of different colors exhibits homologous pairs of different types. Pe-Pe: paralogous pairs of PeTCPs; Pe-Os: orthlogous pairs of TCPs between moso bamboo and rice; Pe-Bd: orthlogous pairs of TCPs between moso bamboo and *Brachypodium distachyon*; Pe-Sb: orthlogous pairs of TCPs between moso bamboo and *Sorghum*.

To explore the evolutionary patterns, divergence and selection pressure of the TCPs, the homologous pairs between moso bamboo and other three grass species (**Supplementary Table S3**), including eight paralogous pairs and 30 orthologous pairs, were identified using phylogeny-based and bidirectional best-hit methods. The *K*a/*K*s ratio is widely applied to measure genetic evolution and selection pressure. According to the natural selection theory, if the ratio is greater than 1, then positive selection is indicated; equal to 1 indicates neutral selection and less than 1 indicates negative or stabilizing selection. Scatter plot statistics showed that PeTCPs have undergone significant negative or stabilizing selection over the course of evolution (**Figure 6**). By contrast, the ratios of two homologous pairs (*PeTCP2/OsTCP1* and *PeTCP10/SbTCP15*) were greater than 1, indicating positive selection during evolution (**Figure 6**). Meanwhile, the divergence times of the *TCP*s were also calculated. The eight paralogous pairs indicated that *PeTCP*s underwent duplication events from ∼5.39 to 58.90 million years ago (MYA). Additionally, the orthologous pairs demonstrated that *PeTCP*s and *OsTCP*s were separated around 19.35 to 55.73 MYA, *BdTCP*s around 22.48 to 59.87 MYA, and *SbTCP*s around 25.78 to 66.55 MYA.

### Tissue-Specific *TCP* Expression Levels of Different Tissues and Development Stages in Moso Bamboo

**FIGURE 7:**
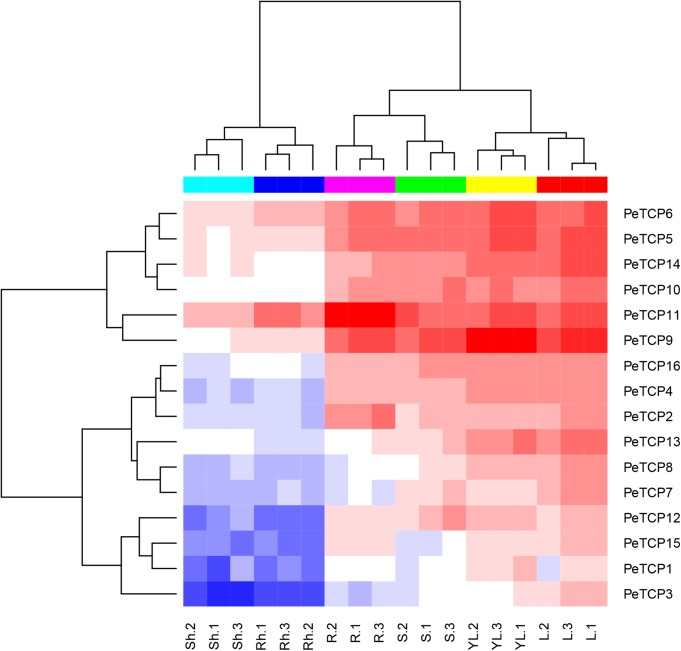
Expression patterns of TCP genes in different tissues and developmental stages of moso bamboo. Lanes: YL, young leaf; L, mature leaf; S, stem; Sh, shoot; Rh, rhizome; R, root. The mean values and SDs were obtained from three biological and three technical replicates.

Gene expression pattern analyses in various tissues and developmental stages can contribute to understanding the roles of genes. To research the expression patterns of *PeTCP* genes in different tissues or developmental stages, including mature leaf, young leaf, stem, shoot, rhizome, and root tissues from the same strain of moso bamboo, the related transcription levels of the *PeTCP* genes were analyzed using qRT-PCR. As exhibited in **Figure 7**, all 16 *PeTCP* genes in the young and mature leaf, 13 genes (except *PeTCP1, 3*, and *15*) in the stem, 12 genes (except *PeTCP1, 7, 8*, and *13*) in root, four genes (*PeTCP5, 6, 9*, and *11*) in rhizome, and four genes (*PeTCP5, 6, 11*, and *14*) in shoot showed high expression levels. In particular, three genes (*PeTCP5, 6*, and *11*) showed high expression levels in all six tissues. In addition, all of the *PeTCP* genes displayed similar expression patterns between young and mature leaves, as well as between shoot and rhizome.

### Subcellular Localization and Transactivation Activity

**FIGURE 8:**
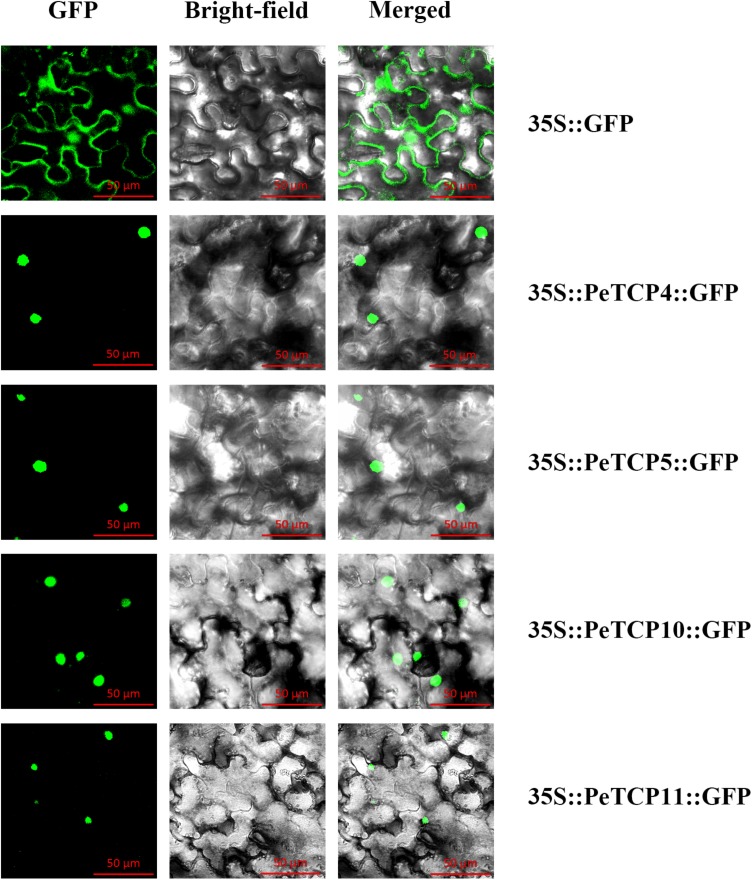
Subcellular localization of four *PeTCPs*. The four PeTCP-GFP fusion proteins (PeTCP4-GFP, PeTCP5-GFP, PeTCP10-GFP, and PeTCP11-GFP) and GFP as a control were transiently expressed in *N. tabacum* leaves and observed under a fluorescence microscope.

In general, TFs can regulate the transcription of target genes by binding to specific *cis*-elements in their promoters and this binding occurs in the nucleus. Based on their characteristics as TFs, *PeTCP*s should localize in the nuclei. To examine this feature, the full-length CDSs without stop codons were cloned from moso bamboo cDNAs using specific primers (**Supplementary Table S4**). Later, they were independently transformed into the pCAMBIA1305 vector containing GFP under the control of the CaMV 35S promoter. The resulting 35S::GFP::PeTCPs and 35S::GFP fusion proteins were subsequently transiently expressed in *Nicotiana tabacum* leaves by *Agrobacterium*-mediated transformation. After 36 h of expression, the leaves of *Nicotiana tabacum* harboring the fusion proteins were observed using confocal laser scanning microscopy (Carl Zeiss LSM710, Germany). GFP fluorescence and light-field observations were recorded in separate channels and then merged into an overlay image. Fluorescence microscopy showed that *PeTCP4, 5, 10*, and *11* were clearly localized in the nucleus according to the GFP signals (**Figure 8**).

**FIGURE 9:**
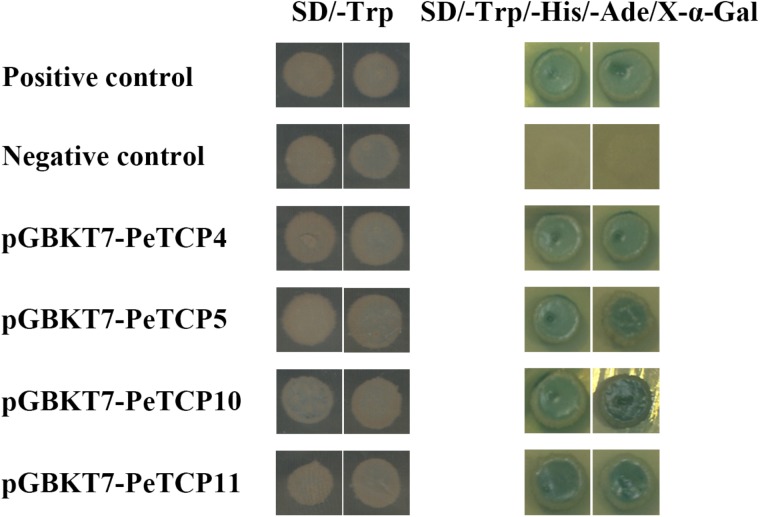
Transactivational analyses of PeTCP proteins in yeast Y2HGold strain. The positive constructs, negative constructs, and fusion constructs were transformed into yeast Y2HGold strain and successively incubated in SD/-Trp media and SD-His/-Ade/-Trp plate supplemented with X-α-GAL.

To explore the transactivation activity of PeTCPs, the pGBKT7::PeTCPs (specific primers listed in **Supplementary Table S5**), the positive control plasmids pGBKT7-53 and pGADT7-T, and the negative control plasmid were independently transformed into the Y2HGold yeast strain. All of these transformants could readily grow and exhibited visible white colonies on the SD/-Trp medium (**Figure 9**). In the SD/-Ade/-His/-Trp/X-α-GAL medium, only the yeast cells containing *PeTCP*s and the positive controls grew well and turned blue. In contrast, the negative control group did not grow on this medium (**Figure 9**). Thus, the four selected *PeTCP* fusion constructs activated the transcription of the *His3* and *LacZ* reporter genes, indicating that they had transcriptional activity in yeast strains.

### Expression Patterns After ABA, Me-JA, and SA Treatment

**FIGURE 10:**
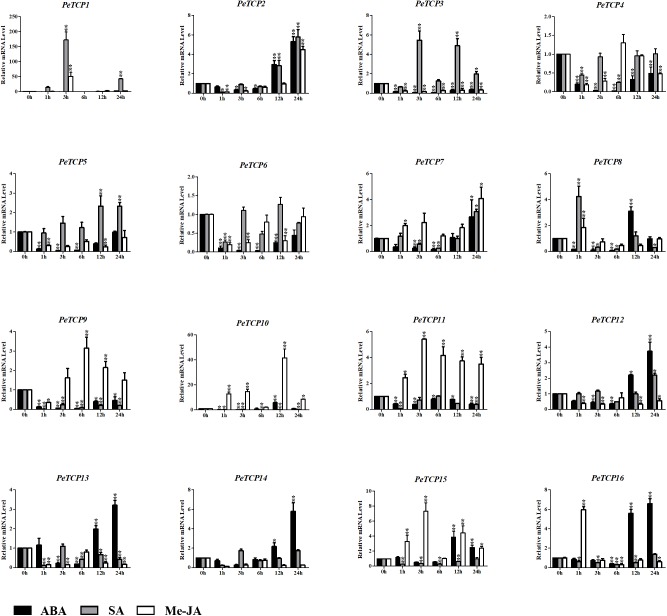
Expression levels of PeTCPs under ABA, Me-JA, and SA treatment by qRT-PCR. The *Y*-axis and *X*-axis indicate the relative expression levels and the time courses of plant hormone treatments, respectively. Error bars, 6 ± SE. Asterisks indicate significant difference compared to the transcription level of control groups, as determined by Student’s *t*-test (^∗^*p* < 0.05, ^∗∗^*p* < 0.01).

Plant hormones, especially ABA, Me-JA, and SA, have well-established roles in plant stress-signaling networks and developmental processes. Many corresponding responsive *cis*-elements have been found in the promoter regions of the *PeTCP*s. Hence, the qRT-PCR test was used to investigate the potential functions of *PeTCP*s in response to ABA, Me-JA, and SA (**Figure 10**). After the ABA treatment, the transcriptional levels of nine *PeTCPs* increased to significantly high levels, three (*PeTCP8, 10*, and *15*) of which were at their highest levels at 12 h and then showed downward trends at 24 h. The remaining members having high transcriptional levels (*PeTCP2, 7, 12–14*, and *16*) showed a downward trend during the early treatment and increased at 24 h. Seven *PeTCP*s exhibited low expression levels during treatment, especially at the early post-treatment stage. After the SA treatment, seven TCPs (*PeTCP1–3, 5, 7–8*, and *12*) were upregulated and compared with control groups, especially *PeTCP1* at 3 h, with expression levels more than 150-fold higher than those of the control group. The remaining *PeTCP* members showed varying degrees of downregulation after the SA treatment. In addition, for the Me-JA treatment, *PeTCP* members also had different expression levels. *PeTCP1–2, 7–11, 15*, and *16* showed an upregulation trend or maintained high expression levels at different time points. In contrast, the expression levels of *PeTCP3–6* and *PeTCP12–14* were lower than in the control groups.

## Discussion

### TCP TFs in Moso Bamboo

The plant-specific TCP TFs with versatile functions in various plant growth and development processes have been functionally analyzed in the model plant species *Arabidopsis thaliana* and rice. Additionally, they have been genome-wide identified in other higher plants, including in dicotyledons such as tea plant (Wu Z.J. et al., 2017), apple (Xu et al., 2014), *Gossypium raimondii* (Ma et al., 2013), watermelon (Shi et al., 2016), *Prunus mume* (Zhou et al., 2016), strawberry (Wei et al., 2016), *Chrysanthemum morifolium* (Wang et al., 2017), and poplar (Ma et al., 2016), and in monocotyledons such as maize (Chai et al., 2017) and *Sorghum* (Francis et al., 2016). Here, 16 TCPTFs were identified in the moso bamboo genome (**Table 1**), and a series of bioinformatics analyses were performed to explore their potential structural and functional characteristics. Compared with other species, moso bamboo contains less TCP members, even though it has the largest genome size (2.075 Gb) (Peng et al., 2013), which may signify that gene loss events occurred over the course of evolution.

The sequence alignment of 16 TCP proteins showed that a non-canonical basic helix–loop–helix motif in the N-terminus is present in the TCP members (**Figure 2**). These members can be divided into two classes based on the presence of four amino acids in the basic motif (Cubas et al., 1999). Class I contains 16 residues, while Class II contains 20 residues. Additionally, two extremely uncharged hydrophilic amino acids, threonine, and glycine, exist in all members of Class II. An arginine-rich R domain outside the conserved TCP domain, with the predicted function of facilitating protein–protein interactions, exists in several class II TCP members (Cubas et al., 1999). For instance, four genes (*OsTCP6, 7, 13*, and *14*) in rice, four (*AtTCP2, 12, 18*, and *24*) in *Arabidopsis thaliana*, maize TB1, and four (*FvTCP3, 6, 9*, and *14*) in *Fragaria vesca* (Wei et al., 2016) contain an arginine-rich R domain according to previous reports. Similarly, *PeTCP13*, the TB1-like gene in moso bamboo, was identified as having an R domain. Thus, the genes containing an R domain may play similar roles in plant growth and development.

However, an intrinsically disordered region exists in the C-terminus of *AtTCP8* and has been identified as a transactivation domain (Valsecchi et al., 2013). Furthermore, similar to reports in *Arabidopsis*, TCP proteins of moso bamboo are also rich in disorder-promoting residues. The transactivation activity experiments with *PeTCP4, 5, 10*, and *11* revealed that these four *PeTCPs* are transcriptional genes in yeast (**Figure 9**).

The TCP domain is a plant-specific DNA-binding domain (Kosugi and Ohashi, 2002), and the members containing this domain regulate the expression levels of other proteins by binding to their promoters. For instance, *PCF2*, which encodes a rice TCP protein, was identified as a DNA-binding protein that recognizes the *PCNA* promoter (Kosugi and Ohashi, 1997). It also acts as a transcriptional activator of *OsNHX1* in salt tolerance by binding to the *OsNHX1* promoter (Almeida et al., 2016). *PeTCP10*, a *PCF2*-like gene, may have a similar binding site and regulatory mechanism as that found in rice.

As DNA-binding proteins, the TCP factors are expected to be targeted to the nuclei. Nuclear localization signals are present in many of them, and nuclear localization has been confirmed for several TCP members by the immunoprecipitation of nuclear extracts or GFP-protein fusions. The four members examined in this study were located in the nucleus (**Figure 8**), consistent with earlier reports (Wei et al., 2016). In contrast, some TCP members were not localized in the nucleus. For example, *FvTCP17* is confirmed to localize in the nucleus and cytoplasm as assessed by the transformation of GFP-protein fusions into *Arabidopsis thaliana* mesophyll protoplasts (Wei et al., 2016).

In addition, the exon/intron and conserved motif distribution patterns of *PeTCP* paralogous genes, such as *PeTCP1/3, 6/11*, and *7/16*, were very similar. These similarities between paralogous pair members might signify their similar functions during moso bamboo growth and development.

### The Potential Functions of TCP TFs in Stress-Related Plant Hormone Transduction in Moso Bamboo

Abscisic acid is produced in both dehydrated vegetative tissues under water deficit conditions and maturing seeds, and it regulates the expression levels of many genes that may function in dehydration tolerance (Yamaguchishinozaki and Shinozaki, 2005). Relationships between the TCPs and ABA have been reported. An earlier report (Mukhopadhyay and Tyagi, 2015) showed that *OsTCP19* which is induced by salt, drought, and cold stresses can improve ABA signal transduction by promoting the expression of ABA INSENSITIVE4 and interacting directly with the encoded protein. In *Arabidopsis thaliana, TCP14* interacts with the DNA BINDING WITH ONE FINGER 6 TF to inhibit the activation of the ABA biosynthetic gene ABA DEFICIENT1 and other ABA-related stress genes to promote the germination of Arabidopsis seeds (Tatematsu et al., 2008; Ruedaromero et al., 2012). ABA-inducible genes contain a conserved ABRE, which functions in the ABA-dependent gene expression induced by osmotic and cold stresses (Yamaguchishinozaki and Shinozaki, 2005). ABRE is a major *cis*-acting element in ABA-responsive gene expression. It exists in the promoter regions of several *PeTCP*s, according to *cis*-element prediction results (**Figure 5**). Additionally, the fluctuations in *PeTCP* gene expression levels were induced after the ABA treatment (**Figure 10**), indicating that these genes may be involved in the ABA signal transduction pathway in moso bamboo through a mechanism similar to that of rice or *Arabidopsis thaliana*.

Meanwhile, TCPs interact with the genes that are involved in the biosynthesis of JA and other oxylipins, which affect development, abiotic stress responses, and plant–microbe interactions. In this pathway, *LOX2*, the best-characterized TCP-controlled gene, encodes a chloroplast enzyme involved in JA synthesis from α-linolenic acid (Vick and Zimmerman, 1983). Its expression is controlled by Class I and Class II TCPs, especially *TCP4*, through an antagonistic mode of action (Schommer et al., 2008). The inactivation of *TCP4* results in *LOX2* downregulation, which reduces JA synthesis and increases plant susceptibility to stress (Sugio et al., 2014). Similarly, the expression patterns of *TCP*s in moso bamboo were diverse after the Me-JA treatment. Additionally, *PeTCP2*, the *TCP4-like* gene, maintains a higher expression level after the Me-JA treatment (**Figure 10**). Thus, the JA regulatory mechanism may be conserved in the TCPs in moso bamboo.

In addition to their functions involving ABA and JA, TCPs also play crucial roles in stimulating the synthesis of, and response to, SA. In *Arabidopsis thaliana*, several TCPs interact with the SA biosynthetic enzyme ISOCHORISMATE SYNTHASE 1 gene, and the gene’s expression is enhanced by the TCPs, such as *TCP8, 9*, and *20*, binding to the TCP-binding motif in its promoter region (Xiaoyan et al., 2015). There are many SA-related *cis*-elements in the promoter regions of *PeTCP*s (**Figure 5**), and the expression levels of several *PeTCP* genes significantly increased after the SA treatment (**Figure 10**), indicating that PeTCPs may have roles in the signal transduction of SA, as reported.

Interestingly, the expression of some genes by SA, Me-JA, and ABA are different from that predicted (**Figure 10**). For example, expression induction of *PeTCP2* has been induced by ABA treatment although ABA-related *cis*-elements are not predicted in the promoter region of *PeTCP2*; expression induction of TCP 10 has been induced by Me-JA treatment although Me-JA-related *cis*-elements are not predicted in the promoter region of *PeTCP10*. On the other hand, expression alteration of *PeTCP10* was not observed by ABA treatment although ABA-related *cis*-elements were detected. Meanwhile, this is observed in TCPs of strawberry (Wei et al., 2016). For example, expression induction of *PvTCP3, -5, -12, -13, -16*, and *-19* have been induced by ABA treatment although ABA-related *cis*-elements are not predicted in their promoter region. These results may show that expression induction of genes are complex biological processes.

## Conclusion

In this study, we investigated the phylogenetics, multiple sequence alignment, gene structures, conserved motifs, *cis*-acting elements, and divergence time analysis of the 16 predicted TCP TFs in the moso bamboo genome. We used qRT-PCR to explore the expression patterns of the 16 *TCP* genes in different tissues and developmental stages, as well as after three – ABA, Me-JA, and SA – plant hormone treatments. Additionally, the subcellular localization and transcription activity analysis of four selected TCP members were investigated in moso bamboo. The results of this study increase the understanding of TCP functions in diverse aspects of plant growth and development.

## Author Contributions

H-LL designed the experiments, carried out the main bioinformatics analyses, and drafted the manuscript. MW and Y-MG participated in the design of the study. FL helped to draft the manuscript. FC implemented the software and helped to draft the manuscript. YX participated in its design and coordination and helped to draft the manuscript. All authors read and approved the final manuscript.

## Conflict of Interest Statement

The authors declare that the research was conducted in the absence of any commercial or financial relationships that could be construed as a potential conflict of interest.

## Footnotes

1 https://trace.ncbi.nlm.nih.gov/Traces/sra/?study=ERP001341

2 http://pfam.sanger.ac.uk/search

3 http://rice.plantbiology.msu.edu/

4 http://www.biomart.org/

5 http://www.arabidopsis.org/

6 https://phytozome.jgi.doe.gov/pz/portal.html

7 http://meme.sdsc.edu/meme/intro.html

8 http://www.physics.csbsju.edu/stats/

9 http://bioinformatics.psb.ugent.be/webtools/plantcare/html/
