# Supplementary material for: TCP Transcription Factors in Moso Bamboo (Phyllostachys edulis): Genome-Wide Identification and Expression Analysis
Source: Front Plant Sci. 2018 Oct 5;9:1263. doi: 10.3389/fpls.2018.01263 (PMC6182085; doi:10.3389/fpls.2018.01263)
Supplement: Supplementary file 1 [file Table_1.DOCX]

Table S1 Information about TCP members in rice, *Brachypodium distachyon*, *Sorghum, Arabidopsis thaliana* and poplar*.*

| Gene Name | Gene ID | Gene Name | Gene ID |
| --- | --- | --- | --- |
| *OsTCP1* | LOC_Os01g11550 | *AtTCP2* | At4g18390 |
| *OsTCP2* | LOC_Os01g55750 | *AtTCP3* | At1g53230 |
| *OsTCP3* | LOC_Os01g69980 | *AtTCP4* | At3g15030 |
| *OsTCP4* | LOC_Os02g42380 | *AtTCP5* | At5g60970 |
| *OsTCP5* | LOC_Os02g51280 | *AtTCP6* | At5g41030 |
| *OsTCP6* | LOC_Os02g51310 | *AtTCP7* | At5g23280 |
| *OsTB1* | LOC_Os03g49880 | *AtTCP8* | At1g58100 |
| *OsTCP8* | LOC_Os03g57190 | *AtTCP9* | At2g45680 |
| *PCF1* | LOC_Os04g11830 | *AtTCP10* | At2g31070 |
| *OsTCP10* | LOC_Os04g44440 | *AtTCP11* | At2g37000 |
| *OsTCP11* | LOC_Os05g43760 | *AtTCP12* | At1g68800 |
| *OsTCP12* | LOC_Os06g12230 | *AtTCP13* | At3g02150 |
| *OsTCP13* | LOC_Os07g04510 | *AtTCP14* | At3g47620 |
| *OsTCP14* | LOC_Os07g05720 | *AtTCP15* | At1g69690 |
| *OsTCP15* | LOC_Os08g33530 | *AtTCP16* | At3g45150 |
| *PCF2* | LOC_Os08g43160 | *AtTCP17* | At5g08070 |
| *OsTCP17* | LOC_Os09g24480 | *AtTCP18* | At3g18550 |
| *OsTCP18* | LOC_Os09g34950 | *AtTCP19* | At5g51910 |
| *OsTCP19* | LOC_Os11g07460 | *AtTCP20* | At3g27010 |
| *OsTCP20* | LOC_Os12g02090 | *AtTCP21* | At5g08330 |
| *OsTCP21* | LOC_Os12g07480 | *AtTCP22* | At1g72010 |
| *OsTCP22* | LOC_Os12g42190 | *AtTCP23* | At1g35560 |
| Bradi1g06460 | Bradi1g06460 | *AtTCP24* | At1g30210 |
| Bradi1g11060 | Bradi1g11060 | *PtrTCP1* | Potri.001G060000.1 |
| Bradi1g45220 | Bradi1g45220 | *PtrTCP2* | Potri.001G111800.1 |
| Bradi1g58450 | Bradi1g58450 | *PtrTCP3* | Potri.001G327100.1 |
| Bradi2g20060 | Bradi2g20060 | *PtrTCP4* | Potri.001G375800.1 |
| Bradi2g50190 | Bradi2g50190 | *PtrTCP5* | Potri.002G152200.1 |
| Bradi2g50687 | Bradi2g50687 | *PtrTCP6* | Potri.003G120200.1 |
| Bradi2g59240 | Bradi2g59240 | *PtrTCP7* | Potri.003G167900.1 |
| Bradi3g36590 | Bradi3g36590 | *PtrTCP8* | Potri.004G046300.1 |
| Bradi3g49660 | Bradi3g49660 | *PtrTCP9* | Potri.004G065800.5 |
| Bradi3g59320 | Bradi3g59320 | *PtrTCP10* | Potri.004G116100.1 |
| Bradi3g60350 | Bradi3g60350 | *PtrTCP11* | Potri.004G222100.1 |
| Bradi4g01550 | Bradi4g01550 | *PtrTCP12* | Potri.005G090300.2 |
| Bradi4g24550 | Bradi4g24550 | *PtrTCP13* | Potri.005G140600.1 |
| Bradi4g29980 | Bradi4g29980 | *PtrTCP14* | Potri.006G125800.1 |
| Bradi4g35520 | Bradi4g35520 | *PtrTCP15* | Potri.008G115800.1 |
| Bradi4g35670 | Bradi4g35670 | *PtrTCP16* | Potri.009G009400.1 |
| Bradi4g41430 | Bradi4g41430 | *PtrTCP17* | Potri.010G130200.1 |
| Bradi5g02880 | Bradi5g02880 | *PtrTCP18* | Potri.011G055500.1 |
| Bradi5g16270 | Bradi5g16270 | *PtrTCP19* | Potri.011G083100.4 |
| *SbTCP1* | Sobic.001G066100 | *PtrTCP20* | Potri.011G096600.2 |
| *SbTCP2* | Sobic.001G121600 | *PtrTCP21* | Potri.012G059900.1 |
| *SbTCP3* | Sobic.002G035500 | *PtrTCP22* | Potri.012G135900.1 |
| *SbTCP4* | Sobic.002G141450 | *PtrTCP23* | Potri.013G110700.1 |
| *SbTCP5* | Sobic.002G198400 | *PtrTCP24* | Potri.013G119400.1 |
| *SbTCP6* | Sobic.002G268600 | *PtrTCP25* | Potri.014G078500.1 |
| *SbTCP7* | Sobic.003G018700 | *PtrTCP26* | Potri.015G050500.1 |
| *SbTCP8* | Sobic.003G299700 | *PtrTCP27* | Potri.015G058800.1 |
| *SbTCP9* | Sobic.003G305000 | *PtrTCP28* | Potri.015G138200.1 |
| *SbTCP10* | Sobic.003G408400 | *PtrTCP29* | Potri.016G074200.1 |
| *SbTCP11* | Sobic.004G225400 | *PtrTCP30* | Potri.016G094800.1 |
| *SbTCP12* | Sobic.004G237300 | *PtrTCP31* | Potri.017G094800.1 |
| *SbTCP13* | Sobic.004G354700 | *PtrTCP32* | Potri.017G112000.1 |
| *SbTCP14* | Sobic.006G025000 | *PtrTCP33* | Potri.019G081800.1 |
| *SbTCP15* | Sobic.006G154000 | *PtrTCP34* | Potri.019G091300.1 |
| *SbTCP16* | Sobic.007G135700 | *PtrTCP35* | Potri.T044100.1 |
| *SbTCP17* | Sobic.007G182101 | *PtrTCP36* | Potri.T146100.1 |
| *SbTCP18* | Sobic.008G172200 |  |  |
| *SbTCP19* | Sobic.009G195000 |  |  |
| *SbTCP20* | Sobic.010G092100 |  |  |
