# Supplementary material for: TCP Transcription Factors in Moso Bamboo (Phyllostachys edulis): Genome-Wide Identification and Expression Analysis
Source: Front Plant Sci. 2018 Oct 5;9:1263. doi: 10.3389/fpls.2018.01263 (PMC6182085; doi:10.3389/fpls.2018.01263)
Supplement: Supplementary file 2 [file Table_2.DOCX]

Table S2 The MEME motif sequences and lengths of TCP genes in moso bamboo.

| Motif | Width | Best possible match |
| --- | --- | --- |
| 1 | 41 | DRHTKVEGRDRRIRMPIICAIRVYQLTDELGHKTDGKTIEW |
| 2 | 30 | MDVAGDAGGGRRPNFPLQLLEKKEDQPCST |
| 3 | 36 | IIAATGTGTIPANFTCLNIPLRTSGSSLSIPAHLRD |
| 4 | 29 | LNAARHEIDKLPPLQFPPQDLMAHLPSSM |
| 5 | 14 | LHYWNFPTNMELIP |
| 6 | 27 | NNLGMVSTAMPYNYTGESWNNSNVHEC |
| 7 | 29 | QLGLGQVGGNGSGGGEGHMGILAALNAYR |
| 8 | 56 | WEQEMQQQQYQQQMAGYTQSQIPGTVWMVPSSNTQAGGGAPVGGGGGSESIWTFPQ |
| 9 | 8 | FGGKDRHE |
| 10 | 29 | GVGVGAGKNGNGPGELQVRKVAPKKNTTK |
