# Supplementary material for: TCP Transcription Factors in Moso Bamboo (Phyllostachys edulis): Genome-Wide Identification and Expression Analysis
Source: Front Plant Sci. 2018 Oct 5;9:1263. doi: 10.3389/fpls.2018.01263 (PMC6182085; doi:10.3389/fpls.2018.01263)
Supplement: Supplementary file 3 [file Table_3.DOCX]

Table S3: Homologous pairs between moso bamboo and other three grass species.

| Paralogous pairs | Ka | Ks | Ka/Ks | Divergence time(Mya) |
| --- | --- | --- | --- | --- |
| *PeTCP1/PeTCP3* | 0.06 | 0.15 | 0.41 | 11.33 |
| *PeTCP4/PeTCP5* | 0.03 | 0.18 | 0.18 | 13.72 |
| *PeTCP6/PeTCP9* | 0.06 | 0.07 | 0.87 | 5.39 |
| *PeTCP6/PeTCP11* | 0.33 | 0.74 | 0.45 | 57.18 |
| *PeTCP7/PeTCP16* | 0.08 | 0.13 | 0.57 | 10.05 |
| *PeTCP8/PeTCP4* | 0.17 | 0.68 | 0.24 | 52.51 |
| *PeTCP9/PeTCP11* | 0.26 | 0.77 | 0.34 | 58.90 |
| *PeTCP12/PeTCP8* | 0.43 | 0.70 | 0.61 | 54.06 |
| Orthologous pairs | Ka | Ks | Ka/Ks | Divergence time(Mya) |
| *PeTCP1/OsTCP10* | 0.09 | 0.25 | 0.37 | 19.35 |
| *PeTCP2/OsTCP1* | 0.86 | 0.72 | 1.19 | 55.73 |
| *PeTCP4/OsTCP12* | 0.14 | 0.41 | 0.33 | 31.75 |
| *PeTCP8/OsTCP5* | 0.06 | 0.36 | 0.17 | 28.01 |
| *PeTCP10/PCF2* | 0.19 | 0.35 | 0.55 | 26.83 |
| *PeTCP11/OsTCP11* | 0.26 | 0.38 | 0.70 | 29.16 |
| *PeTCP13/TB1* | 0.54 | 0.59 | 0.91 | 45.76 |
| *PeTCP14/OsTCP8* | 0.30 | 0.39 | 0.77 | 29.97 |
| *PeTCP15/OsTCP3* | 0.27 | 0.44 | 0.61 | 33.91 |
| *PeTCP16/OsTCP18* | 0.33 | 0.36 | 0.92 | 27.38 |
| *PeTCP1/Bradi5g16270* | 0.15 | 0.29 | 0.50 | 22.48 |
| *PeTCP2/Bradi2g50190* | 0.35 | 0.55 | 0.63 | 42.69 |
| *PeTCP6/Bradi2g50687* | 0.25 | 0.61 | 0.41 | 47.27 |
| *PeTCP8/Bradi3g59320* | 0.19 | 0.67 | 0.29 | 51.38 |
| *PeTCP10/Bradi4g35670* | 0.26 | 0.44 | 0.60 | 33.85 |
| *PeTCP11/Bradi2g20060* | 0.26 | 0.73 | 0.36 | 56.07 |
| *PeTCP13/Bradi1g11060* | 0.46 | 0.78 | 0.59 | 59.87 |
| *PeTCP14/Bradi1g58450* | 0.32 | 0.62 | 0.51 | 47.97 |
| *PeTCP15/Bradi2g59240* | 0.10 | 0.36 | 0.28 | 27.91 |
| *PeTCP16/Bradi4g35520* | 0.12 | 0.53 | 0.23 | 40.45 |
| *PeTCP2/SbTCP8* | 0.15 | 0.45 | 0.33 | 34.27 |
| *PeTCP4/SbTCP20* | 0.09 | 0.64 | 0.15 | 49.46 |
| *PeTCP5/SbTCP20* | 0.10 | 0.55 | 0.19 | 42.16 |
| *PeTCP8/SbTCP12* | 0.05 | 0.46 | 0.11 | 35.00 |
| *PeTCP9/SbTCP9* | 0.17 | 0.39 | 0.42 | 30.10 |
| *PeTCP10/SbTCP15* | 0.83 | 0.80 | 1.04 | 61.68 |
| *PeTCP10/SbTCP17* | 0.18 | 0.58 | 0.31 | 44.56 |
| *PeTCP11/SbTCP19* | 0.15 | 0.34 | 0.45 | 25.78 |
| *PeTCP13/SbTCP2* | 0.41 | 0.87 | 0.48 | 66.55 |
| *PeTCP14/SbTCP1* | 0.14 | 0.39 | 0.37 | 29.85 |
