# Supplementary material for: TCP Transcription Factors in Moso Bamboo (Phyllostachys edulis): Genome-Wide Identification and Expression Analysis
Source: Front Plant Sci. 2018 Oct 5;9:1263. doi: 10.3389/fpls.2018.01263 (PMC6182085; doi:10.3389/fpls.2018.01263)
Supplement: Supplementary file 4 [file Table_4.DOCX]

Table S4. Specific primers of four PeTCPs for subcellular localization experiment.

| Name | Sequence |
| --- | --- |
| PeTCP4-F | GCTCTAGAATGGACGTCGCCGGAGACGCC |
| PeTCP4-R | CGGGATCCCGAATCGCTGGCGCTCATGCT |
| PeTCP5-F | GCTCTAGAATGGACGTCGCTGGAGACGCC |
| PeTCP5-R | CGGGATCCCGAGTCGCTGGCGTT |
| PeTCP10-F | GGACTAGTATGGAGGCGCAGGTGCAG |
| PeTCP10-R | TCCCCCGGGCCGGTGGCCGAGA |
| PeTCP11-F | GGACTAGTATGATAAGCGGCAACCAC |
| PeTCP11-R | CGGGATCCCTGGCTTCCCGAGTGAAA |
