# Supplementary material for: TCP Transcription Factors in Moso Bamboo (Phyllostachys edulis): Genome-Wide Identification and Expression Analysis
Source: Front Plant Sci. 2018 Oct 5;9:1263. doi: 10.3389/fpls.2018.01263 (PMC6182085; doi:10.3389/fpls.2018.01263)
Supplement: Supplementary file 5 [file Table_5.DOCX]

| Name | Sequence |
| --- | --- |
| PeTCP4-F | GGAATTCATGGACGTCGCCGGAGACGCC |
| PeTCP4-R | CGGGATCCCTACGAATCGCTGGCGCTCATGC |
| PeTCP5-F | GGAATTCATGGACGTCGCTGGAGACGCC |
| PeTCP5-R | CGGGATCCCTACGAGTCGCTGGCGTT |
| PeTCP10-F | GGAATTCATGGAGGCGCAGGTGCAG |
| PeTCP10-R | GCGTCGACCTACCGGTGGCCGAGAC |
| PeTCP11-F | GGAATTCATGATAAGCGGCAACCACG |
| PeTCP11-R | CGGGATCCACTCTGGCTTCCCGA |

Table S5. Specific primers of four PeTCPs for transcription activity experiment.
